# Bacillus Calmette-Guérin (BCG) Revaccination and Protection Against Tuberculosis: A Systematic Review

**DOI:** 10.7759/cureus.56643

**Published:** 2024-03-21

**Authors:** Adewale Lawrence

**Affiliations:** 1 Pharmaceutical Medicine, Bioluminux Clinical Research, Naperville, USA

**Keywords:** systematic review, mycobacterium tuberculosis, bacillus calmette-guérin (bcg), tuberculosis, revaccination

## Abstract

Bacillus Calmette-Guérin (BCG) vaccination remains a cornerstone in global efforts to combat tuberculosis (TB), a persistent public health threat worldwide. The purpose of this systematic review is to find out how well BCG revaccination protects against TB. This systematic review synthesized recent studies investigating the efficacy of BCG vaccination in preventing TB infection and disease. A total of 15 relevant publications were identified through a comprehensive search across multiple databases, including Cochrane Library, PubMed, Medline, and Scopus. The inclusion criteria encompassed studies involving humans, written in English, and categorized as case-control, cohort, meta-analysis, or full-text. Studies were selected based on their relevance to BCG revaccination and protection against TB, and a standardized data extraction form was used to gather pertinent information from each study. Quality assessment was conducted using established tools to evaluate the rigor, study design, and risk of bias in each included study. The findings revealed significant insights into BCG's effectiveness across different populations and age groups. Several studies demonstrated a substantial reduction in latent TB infection (LTBI) and incidence rates of TB following BCG vaccination. However, the protective efficacy of BCG revaccination varied across studies and populations, with some indicating modest protection against TB disease development, particularly in high-risk populations like healthcare workers. Furthermore, investigations into the immunological mechanisms underlying BCG's protective efficacy provided valuable insights into cytokine/chemokine profiles and immunomodulatory properties.

## Introduction and background

*Mycobacterium tuberculosis* (*M. tuberculosis*) is the bacterium that causes tuberculosis (TB) and is responsible for most cases of TB. Other infectious diseases caused by members of the *M. tuberculosis* complex include Buruli ulcer which is a skin infection caused by *M. ulcerans* and bovine TB, caused by *M. bovis*, which shares symptoms with TB but is usually resistant to pyrazinamide, an antibiotic commonly used to treat TB. According to experts, it ranks as the ninth leading killer on a global scale. A lifetime incidence of TB illness is estimated to be between 5% and 15%, and the expected number of persons afflicted with the disease surpasses 1.7 billion. In the year 2016, it was predicted that approximately 10.4 million individuals worldwide contracted TB. Among these cases, adults accounted for 90%, men accounted for 64%, and individuals living with human immunodeficiency virus (HIV) constituted 9% of the total TB incident cases. The latter population exhibits an elevated susceptibility to TB infection, with an estimated risk ranging from 16 to 27 times higher compared to individuals who are HIV-negative. According to Bouzeyen and Javid, it ranks as the ninth leading killer on a global scale with BRICS (i.e., Brazil, Russia, India, China, and South Africa) countries accounting for 53% of cases. An estimated 10.4 million people fell ill with TB in 2016, with drug-resistant TB being a persistent threat. In 2016, 490,000 cases of multidrug-resistant TB (MDR TB) occurred, and 110,000 cases of rifampin-resistant, isoniazid-susceptible TB occurred [1]. If left untreated, the lifetime chance of developing active TB ranges from 5% to 10%. This progression occurs when the body's immune system becomes compromised, typically months or years after the initial infection. Young children, those with diabetes, and individuals living with HIV are considered to be the most susceptible populations with an increased likelihood of developing active TB disease. Timely identification and intervention are necessary for individuals with latent TB infection (LTBI), especially in high-risk populations like those who are also infected with HIV [2].

TB is best treated with a combination of medications over long periods, according to the available evidence. However, with the rise of HIV and drug-resistant TB (MDR TB), TB has risen to the status of a significant global public health concern. Multiple social and biological factors, such as poverty and insufficient shelter, contribute to the spread of TB among susceptible people. Biological factors include HIV and the development of MDR TB. Furthermore, economic inequality and rapid urbanization are structural factors that significantly contribute to TB transmission [3]. The Bacillus Calmette-Guérin (BCG) vaccine is a single-visit preventive measure against TB in newborns. It is an attenuated strain of *M. bovis*, developed in France in 1921 by Albert Calmette and Camille Guérin. BCG's mechanism of protection is to reduce the spread of bacilli from the primary infection site. However, multiple studies have shown conflicting results, ranging from 80% to negative effects, due to methodological differences, strain variations, and high non-tuberculous cases [4].

The BCG vaccination, produced from *M. bovis*, has been employed worldwide since 1921 to prevent human TB. The vaccine is extensively utilized, with an estimated annual administration of around 100 million immunizations to neonates. BCG vaccination is believed to decrease the transmission of *M. tuberculosis* into the bloodstream from the initial infection site, which can lead to severe conditions such as military TB and TB meningitis [5]. The wide efficacy range of the BCG vaccine is attributed to genotypic differences within the vaccine. The vaccine was first produced by passing a pathogenic *M. bovis* strain over 230 times, leading to its attenuation. Recent genomic sequencing has revealed 16 different regions of differentiation (RDs) in the world's supply of BCG vaccines. Some preliminary data suggests different strains produce differing immunological responses, with some studies showing higher levels of TB drug resistance later in life. Neonates and infants have an actively changing immune system during the first 24 months of life and show multiple innate and adaptive immune limitations that affect the ultimate outcome in immunity upon infection or vaccination [6].

Revaccination of the BCG vaccine can enhance immunity. Nevertheless, the tuberculin response does not correlate with the protective advantages obtained by BCG vaccination. Furthermore, there is a lack of empirical support indicating that a decline in tuberculin sensitivity over time corresponds to a decline in TB immunity. Currently, no efficacious vaccine is available for the prevention of TB in adults, both before and after *M. tuberculosis* infection [7]. An investigation in Kenya, Zambia, South Africa, and Tanzania revealed a 54% safeguard against pulmonary TB sickness in patients infected with *M. tuberculosis*. BCG revaccination continues to be employed in specific areas with high TB prevalence. However, the World Health Organization (WHO) advises against administering subsequent doses to those who have already undergone BCG vaccination [8].

The BCG vaccine does not always protect people from getting TB. The vaccine's efficacy is suboptimal and clearly not adequate for disease control. It inhibits several arms of the innate immune response, including phagosome maturation or cytokine production. The BCG vaccine, or Bacille Calmette-Guérin vaccine, protects against TB disease and mortality in some populations. However, it doesn't prevent primary infection or reactivation of latent pulmonary [9].

## Review

Review methods

The procedures used to search for and evaluate the papers included in this systematic review were in complete accordance with the Preferred Reporting Items for Systematic Reviews and Meta-Analyses (PRISMA) standards laid forth by Liberati et al. [10]. ProQuest, PubMed, and ScienceDirect were the three databases used for this investigation. Using the terms "BCG Revaccination" and "BCG Protection Against Tuberculosis" helped refine the search results. The electronic search was likely relevant to this inquiry. We took great care to exclude any items directly copied from another source or written in a language other than English throughout the screening process. When assessing the articles for the inquiry, we looked at their titles, abstracts, research styles, and how easily we could acquire their entire texts. After sifting through 31 articles in the first search across all three databases, a final tally of 15 papers was chosen. Applying targeted keywords and reviewing abstracts further reduced the number of publications that needed evaluation. Using the inclusion and exclusion criteria, 15 publications were found relevant to the topic at hand [10].

Selection criteria

The review included studies involving humans, written in English, and categorized as either case-control, cohort, meta-analysis, or full-text. This study used grey literature to find fresh information on BCG revaccination and protection against TB. We excluded the research that omitted systematic reviews. We also did not include duplicates or items missing key parts of material.

Search and selection of database

The study utilized various electronic databases, including ScienceDirect, Scopus, PubMed, and Cochrane, to conduct a search strategy. Titles and abstracts were screened for relevance to the review question and inclusion criteria, and highly relevant studies were selected through full-text screening.

Extraction of data

The study utilized a standardized data extraction form to gather pertinent information from selected studies, focusing on research characteristics like design, year, objective, and findings.

Quality assessment

**Figure 1:**
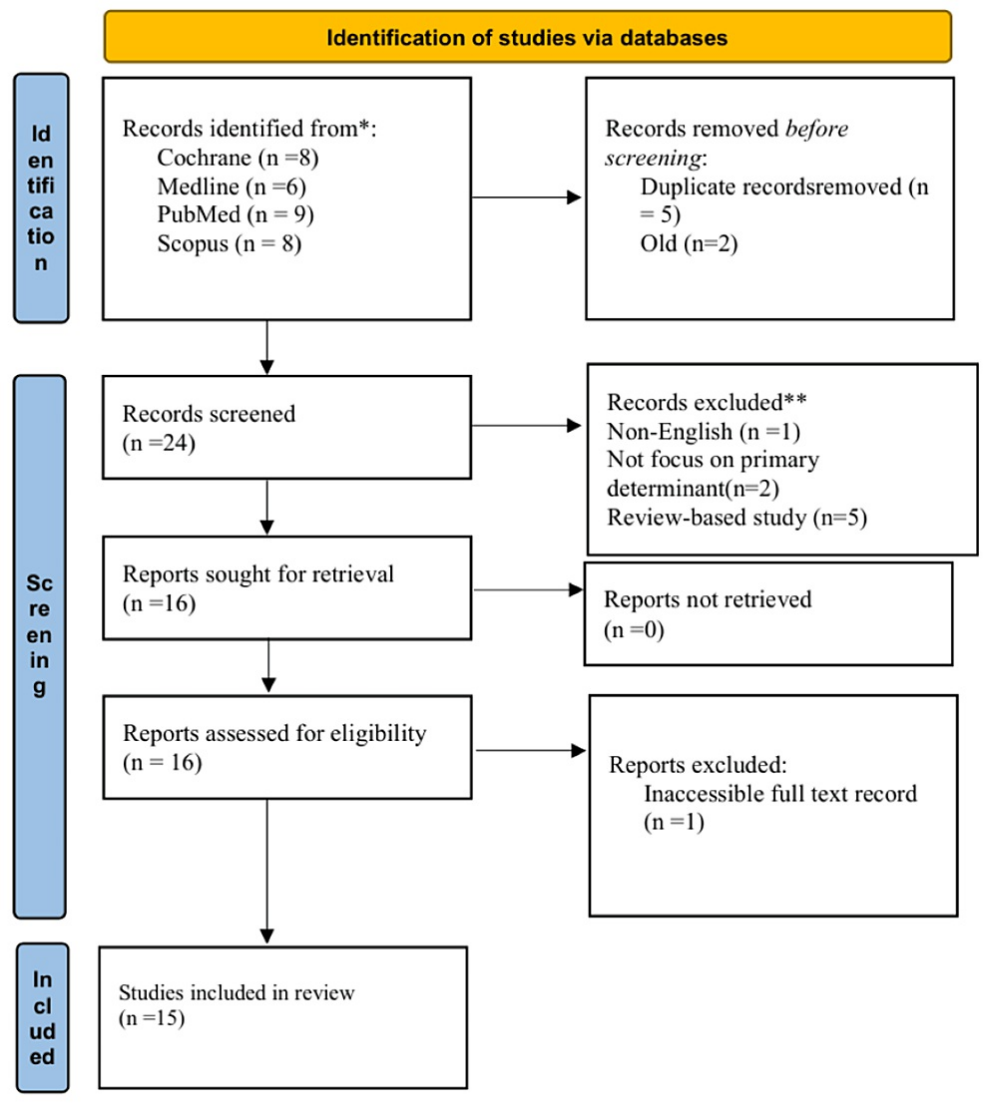
Flowchart of data inclusion strategy for systematic review

The PRISMA (Figure 1) is utilized for the systematic review to ensure that all studies are reported fairly and thoroughly. Researchers used McMaster University's Critical Review Form for Qualitative Studies by Letts et al. [11], the Critical Appraisal Skills Programme by Singh [12] for systematic reviews, or the Guidelines for Critical Review Form: Qualitative Studies by Letts et al. [13] to assess the study's quality. The included studies were evaluated using these quality criteria based on their rigor, study design, and bias risk. The study utilized relevant tools to evaluate the quality of studies, risk of bias, methodological quality, and potential sources of bias in each study.

Data synthesis and analysis

The data was analyzed narratively, systematically organizing outcomes related to BCG revaccination's effectiveness in protecting against TB. Sensitivity and subgroup analyses were performed as needed.

Interpretation of outcomes

The study analyzed the findings, highlighting their limitations and strengths, and discussed their practical implications, potential knowledge gaps, and suggestions for future research.

Report write-up

The manuscript adhered to the PRISMA model guidelines and was organized into five sections: Introduction, Methodology, Results, Discussion, and Conclusion.

Results

Eight articles came from the Cochrane Library, nine from PubMed, six from Medline, and eight from Scopus; the search produced 31 articles. The articles that did not make the cut were duplicates (5), outdated studies (2), written in a language other than English (1), without a primary focus on the essential variables (2), and based on the review-based study (5). One full-text record was unavailable throughout the evaluation. Consequently, fifteen publications were evaluated for appropriateness and ultimately chosen for the study. Each paper was carefully reviewed by looking at its abstract, title, and research kind and whether or not the entire text was available. Table 1 gives the detailed explanations of the studies that were not included in the review on the BCG revaccination and protection against TB. The reasons for the exclusion of these research are also disclosed. These exclusions include studies that are based on reviews and systematic reviews, as well as studies that are out of date, irrelevant to the findings of the research, or focused on themes that are unrelated to the study, such as the degree to which the use of BCG is successful in controlling the spread of coronavirus disease 2019 (COVID-19). An emphasis was placed on current and relevant research that was directly connected to the effect of BCG revaccination and protection against TB. The criteria for exclusion were implemented in order to guarantee the relevance and quality of the studies that were included in the review.

**Table 1:** A summary of the excluded studies BCG: Bacillus Calmette-Guérin; SARS-CoV-2: severe acute respiratory syndrome coronavirus 2; COVID-19: coronavirus disease 2019; MTBVAC: *Mycobacterium tuberculosis* vaccine

Author/year	Objective	Reason for exclusion
Khandelia et al., 2023 [14]	An overview of the BCG vaccine and its future scope	Review
Khademi et al., 2018 [15]	Multi-stage subunit vaccines against *Mycobacterium tuberculosis*: an alternative to the BCG vaccine or a BCG-prime boost?	Review
Glisic et al., 2020 [16]	Biological rationale for the repurposing of BCG vaccine against SARS-CoV-2	Irrelevant outcomes report findings associated with the effectiveness of using BCG for the control of COVID-19 spread
Su et al., 2023 [17]	Progress on specific and non-specific immune effects of BCG	Non-English
Clark et al., 2017 [18]	Revaccination of guinea pigs with the live attenuated MTBVAC improves BCG's protection against tuberculosis	Irrelevant outcomes report
Strapagiel et al., 2008 [19]	Monocyte response receptors in BCG-driven delayed-type hypersensitivity to tuberculin	Old study
Barreto et al., 2006 [20]	BCG vaccine: efficacy and indications for vaccination and revaccination	Old study and Inaccessible full-text record
Bannister et al., 2021 [21]	The safety of BCG revaccination: a systematic review	Systematic review-based study
Machlaurin et al., 2019 [22]	Health economic evaluation of current vaccination strategies and new vaccines against tuberculosis: a systematic review	Systematic review-based study
Pereira et al., 2007 [23]	BCG vaccine against tuberculosis: its protective effect and vaccination policies	Old and systematic review-based study

Table 2 presents a summary of the studies.

**Table 2:** A summary of the studies LTBI: latent tuberculosis infection; BCG: Bacillus Calmette-Guérin; TB: tuberculosis; COVID-19: coronavirus disease 2019; RRR; relative risk reduction; TST: tuberculin skin test; FP: fusion protein; ECST: ESAT6-CFP10 skin test; PBMC: peripheral blood mononuclear cells

Author/year	Objective	Results
Trollfors et al., 2021 [24]	This research aimed to quantify LTBI among immigrant youth in Sweden and assess the efficacy of the BCG vaccine in preventing LTBI.	An estimated 59% of immigrant children without a BCG scar and 17% of those with a scar had LTBI, according to the results. There was a significant decrease in LTBI after receiving the BCG vaccination, suggesting that the virus may spread among immigrants and the general public.
Ręka et al., 2020 [25]	This study's overarching goal is to provide the current level of understanding on how the TB vaccine BCG affects the death and incidence rates caused by COVID-19.	Famous for its immunomodulatory properties, the TB vaccination protects against severe cases of the disease and others. Incidence and fatality rates of COVID-19 are lower in countries that mandate BCG immunization than those that do not.
Bennasrallah et al., 2019 [26]	The purpose of this research was to examine the 18-year TB incidence and trend data, as well as the effects of the BCG vaccine after 40 years of vaccination.	According to the research, there was a 65.1% RRR for pulmonary localization and a 65% RRR for other localizations when administering BCG immunization. With an RRR of 96.7% in patients with pulmonary TB and 86% in patients with other localizations of TB, respectively, Protocol 3 demonstrated the most significant success. According to the research, results showed that BCG vaccination improved TB localizations other than lymph nodes but had the opposite effect on pulmonary TB.
Pelzer et al., 2022 [27]	This research looked at the link between the BCG vaccine given to babies at birth and the infection of *M. tuberculosis*.	While the correlation between BCG scar and TST positivity did not always revert to its positive state, it did decrease as TST cut-off values increased. In contrast to research conducted in Tanzania, the one conducted in Vietnam did not find that BCG vaccination decreased the incidence of *M. tuberculosis* infections.
Martinez et al., 2022 [28]	This research aimed to determine if the BCG vaccine had a protective effect against TB in infants of different ages.	Results showed that BCG immunization substantially protected against TB in children under five. Nevertheless, adults and teenagers do not benefit from it. Immunoprotection should be enhanced in the elderly. Vaccination with BCG was 18% successful in the end.
Mansury et al., 2019 [29]	This research examined the immunogenicity of liposomes containing DDA/TDB and the *M. tuberculosis* FP.	The results showed that the DDA/TDB/CHOL liposomes containing the FP had the highest IFN-γ and IL-12 interleukin. The DDA/TDB/CHOL/FP vaccination was more effective after the first injection of BCG; nonetheless, the groups who got BCG initially had the most robust Th1 response.
Huang et al., 2022 [30]	Our objective was to determine whether the BCG vaccine can prevent *M. tuberculosis* infection in first-year college students using either the TST or the recombinant fusion protein ECST.	ECST may fail to recognize the full protective impact of the BCG vaccine against LTBI in first-year college students despite the vaccine providing excellent protection even in regions with a slightly elevated incidence.
Sweeney et al., 2019 [31]	This 13-year research examines the effect of various BCG vaccination strategies on TB cases in the southern region of Ireland.	This research adds to the current data on TB by showing that identical people in different regions have different infection rates. This is particularly true in low-incidence nations like Ireland, showing that the BCG vaccine effectively prevents primary TB illness.
Dos Santos et al., 2024 [32]	A reevaluation of the TB vaccine *M. bovis* BCG has shown that it is more effective when given at different times and with different approaches.	There must be more about BCG's immunological response because of a lack of research. Future research should fill these gaps to enhance vaccination efficacy and comprehension of BCG's effectiveness.
Kousha et al., 2021 [33]	This research investigated the effects of BCG vaccination history to determine whether *M. tuberculosis* Beijing or non-Beijing strains are more easily prevented.	Vaccination history was strongly correlated with Beijing strains, and there was a statistically significant correlation between Beijing strains and TB drug resistance in isolates. Beijing strains were statistically associated with drug-resistant TB in vaccinated individuals but not those without a vaccination history.
Roy et al., 2019 [34]	Using household surveys, researchers in 152 nations with a high burden of disease estimated BCG coverage by age group.	Research shows that there would be a 2.8% (5449) decrease in TB fatalities among children worldwide if 92% coverage were implemented at birth. Still, despite expanded coverage, TB mortality rose throughout the subsequent government. The research found that if there were fewer delays and more coverage at birth, the death rate from pediatric TB would drop dramatically all over the world.
Katelaris et al., 2020 [35]	We investigated if there is any evidence that the association between BCG vaccination and reduced adult *M. tuberculosis* infection rates persists.	A study in the United Kingdom found that BCG vaccination is associated with a decreased prevalence of LTBI in adults.
Owusu et al., 2023 [36]	This study aimed to evaluate the effectiveness of "BCG-Vaccinated Children with Contact to Tuberculosis Patients Show Delayed Conversion of Mycobacterium tuberculosis-Specific IFN-γ Release."	The study found that the immune system's ability to recognize *M. tuberculosis* antigens happens earlier in kids and teens who haven't been vaccinated against BCG. This could be because of a higher threshold for T-cell activation or other factors that are important during early *M. tuberculosis* infections and delay T-cell conversion in people who have been vaccinated against BCG.
Velayutham et al., 2023 [37]	This study aimed to reassess the Chingleput BCG vaccination trial, conducted in 1968, for the protective efficacy of BCG revaccination against incident TB disease.	The retrospective analysis of a community-based trial found that BCG revaccination provided modest protection against TB disease development after 15 years, but further evaluation is needed.
Subbian et al., 2020 [38]	A study compared the cytokine/chemokine profiles of PBMC from infants vaccinated at birth to those before BCG vaccination.	Results showed that BCG-vaccinated infants released higher levels of pro-inflammatory molecules and displayed distinct pro- and anti-inflammatory cytokine/chemokine patterns when exposed to different *M. tuberculosis* strains.

The study by Trollfors et al. investigated the prevalence of LTBI in immigrant children and the effectiveness of BCG against LTBI in Sweden. The study involved 1,404 immigrants aged 0-17 years, with a BCG scar used as a substitute for written documentation. Results showed LTBI was common among immigrant children (17%), and if not tested and treated, it could spread to the immigrant population and the general population [24].

Ręka et al.'s study examined the impact of the BCG vaccine against TB on COVID-19 incidence and death. PubMed, ResearchGate, and WHO reports from April and May 2020 were used. Countries with mandated BCG immunization had lower COVID-19 incidence and death than those without. The BCG vaccine induces heterologous immunity and trained innate immunity. Genetics, strain kind, healthcare level, nation's affluence, migratory structure, comorbidities, and social distance policies may also affect the link. A reduction in COVID-19 incidence and death in nations with mandatory BCG immunization needs more study, the study found [25].

Bennasrallah et al.'s study analyzed the incidence and trends of TB over 18 years in Monastir, Iran. The study found that pulmonary localization was the predominant site of TB, with a relative risk reduction (RRR) of 65.1% for pulmonary localization and 65% for other localizations. Protocol 3 (at birth) had the highest effectiveness, with an RRR of 96.7% and 86% for patients with pulmonary TB and other localizations, respectively. The study concluded that the BCG vaccine had a positive impact on pulmonary TB and other TB localizations, but had a reverse effect on lymph node TB [26].

Pelzer et al. reported a favorable correlation between birth BCG vaccination and *M. tuberculosis* infection in Vietnamese schoolchildren. As tuberculin skin test (TST) cut-off values increased, the correlation weakened but never flipped. The study suggests that geographical variances and Vietnam's high Beijing genotype prevalence may explain the discrepancies [27].

Martinez et al. examined the age-specific effects of baby BCG immunization on TB development and mortality in a comprehensive review and meta-analysis. The study indicated that BCG immunization prevented TB in young children but not adolescents or adults. The study indicated that BCG immunization only significantly prevented all TB in children under five. All participants were protected from pulmonary TB but not extrapulmonary. In four mortality studies, BCG immunization dramatically reduced mortality. The data imply that BCG immunization at birth prevents TB in young children but not adolescents or adults. The immune defense must be increased in older people [28].

Mansury et al. studied that TB is a global health issue and developing an effective vaccine is crucial. The study evaluated the immunogenicity of *M. tuberculosis* fusion protein (FP) encapsulated in liposomes containing DDA/TDB. Results showed the highest IFN-γ and IL-12 interleukin concentrations in the FP-containing liposomes. Initial injection with BCG improved the vaccine's efficacy, and the groups receiving BCG and then DDA/TDB/CHOL/FP had the greatest Th1 response [29].

The study by Huang et al. investigated the effectiveness of BCG in protecting first-year college students against *M. tuberculosis* infection. A cross-sectional study found that BCG was significantly associated with LTBI, especially in rural areas. The study suggests that a novel skin test may underestimate BCG's protective effects and that BCG offers better protection in areas with a slightly higher incidence of LTBI [30].

Sweeney et al. examined the impact of different BCG vaccination policies on the incidence of TB disease in the southwest of Ireland over 13 years. Results show significant regional variation in TB incidence in demographically similar populations based on BCG vaccination policy, particularly in a country with low TB disease incidence, such as Ireland [31].

Dos Santos et al. found that a BCG-Denmark immunization research for adult pulmonary TB in Brazilian healthcare workers reported no reduction in first QFT Plus conversion risk. A nested randomized controlled experiment was conducted inside the BRACE trial, including Brazilian healthcare professionals aged ≥18. The main effectiveness endpoint was QFT Plus conversion (≥0.35 IU/mL) 12 months after immunization for patients with a negative baseline. The findings suggest that high-risk communities require greater TB preventive knowledge. BCG-Denmark immunization may protect adolescents and adults against TB, although its effectiveness in adults is unclear [32].

Kousha et al. compared the effect of BCG vaccination history on preventing *M. tuberculosis* Beijing and non-Beijing strains. It found a significant relationship between Beijing strains and TB drug resistance and a strong association between vaccination history and Beijing strains. Further investigation is needed with a larger sample size [33].

Roy et al. modeling study found that delayed BCG vaccinations are widespread in countries with high TB burdens. The study estimated age-specific BCG coverage in 152 high-burden countries and found that achieving 92% coverage at birth reduced TB deaths by 5449 or 2.8% by age 15 years. However, later administration increased TB deaths, even with increased coverage. The study recommends maintaining the WHO recommendation for BCG at birth [34].

Katelaris et al. conducted a cohort study and found that BCG vaccination reduces *M. tuberculosis* infection in adults, with a 30% vaccine effectiveness against LTBI. This suggests BCG may protect against the disease, impacting immunization programs, vaccine development, and global TB control efforts [35]. 

Owusu et al.'s study suggested findings that the immune system's ability to recognize *M. tuberculosis* antigens happens earlier in kids and teens who haven't been vaccinated against BCG. This could be because of a higher threshold for T-cell activation or other factors that are important during early *M. tuberculosis* infections and delay T-cell conversion in people who have been vaccinated against BCG [36].

Velayutham et al. re-analyzed the Chingleput BCG vaccination trial to assess its protective efficacy against incident TB disease. Results showed that BCG revaccination offered modest protection against TB disease at 15 years post-vaccination, but further evaluation is needed. The study highlights the importance of BCG vaccination in TB prevention [37].

Subbian et al. revealed that the protective immunity elicited by BCG vaccination against TB is highly variable and poorly understood. It also found that BCG-induced infant immune responses and their potential protective capacity may be shaped by the nature of the infecting *M. tuberculosis* strain [38].

Discussion

Results from previous research that followed up with 4436 people for 15 years after their immunization showed that BCG revaccination provided 36% protection against TB [38]. Additionally, prior research indicated that BCG revaccination protected men but did not protect females. Research has shown that BCG has distinct effects on men and women. Research shows a gender difference in the preventive effects of newborn BCG vaccination on all-cause mortality and morbidity, with boys showing a high protective impact during the first week and weak benefits after two weeks [39]. People in the age bracket of 31-40 years old at the time of trial intake were the only ones shown to have a protective effect against TB with BCG revaccination. This calls for further research into the optimal intervals between BCG revaccinations to prevent TB [40]. Previous research has shown that revaccination with BCG produces a positive immune response. A recent South African phase II clinical study for TB infection prevention in adolescents found that BCG revaccination reduced the incidence of sustained quantifier TB (QFT) conversion by 45.4%. Therefore, there is a need for more research on the efficacy of BCG revaccination in halting the course of TB infections [41]. Possible causes for the varying levels of protection provided by a single BCG treatment include pre-sensitization with environmental mycobacteria, differences in BCG strain, area of administration, and geographical location. There was no correlation between the strain of BCG and its effectiveness against TB, and a comprehensive study found that a greater level of BCG efficiency was connected with a lack of history of infection or sensitization to environmental mycobacteria [42].

When compared to new vaccination choices, BCG has inherent advantages due to the fact that it has been used for a long time and has a health system that is optimized for vaccine distribution. Revaccination with BCG has been subjected to extensive research and has been shown to have a moderate level of safety. On the other hand, a study conducted in Australia discovered that revaccinated individuals had a greater frequency of abscess and lymphadenopathy, both of which resolved within a month if they were not treated [43]. Prior research also attempted to compare the duration of TB breakdown in the BCG and placebo groups but found no statistically significant differences between the groups up to five or 10 years after vaccination. Further review in prospective research is necessary to fully assess the results; however, they are beneficial for idea formulation. There is a lack of information about possible confounding factors, including dietary habits, socioeconomic status, and TB exposure status [44]. After 15 years of follow-up after immunization, the prior research found that BCG revaccination provides 36% protection against TB. To find out how well this research worked in India, more assessment is required [45].

Aiming to investigate the effectiveness of BCG revaccination against TB in school-aged children aged 7-14 years, Rodrigues et al. conducted research in two Brazilian towns, Salvador and Manaus. Tuberculosis Control Programme Surveillance System data linking allowed for the identification of TB cases. Nine percent of TB cases were successfully treated with BCG revaccination. The research found no increased protection for children vaccinated between the ages of seven and 14 [46].

A study in northern Malawi found that a second BCG vaccination increased the incidence rate ratio of diagnostically definite TB in scar-positive individuals compared to placebo scar-positive individuals; none of the experimental vaccinations protected pulmonary TB. The study suggests that a second vaccination could significantly increase leprosy protection without TB protection [47].

A longitudinal study in Finland examined the effectiveness of BCG revaccination in preventing TB in Finnish students. Six groups of people were included, and six years after the program's termination, three TB cases were reported among children who had not received the vaccine. The study found no correlation between the end and TB cases [48]. Children aged six to nine in Hong Kong were the subjects of an investigation on the efficacy of a BCG immunization program. From 1978 to 1982, a BCG revaccination program was run, with 258,700 participants. There was no statistically significant change in tuberculin incidence rates, indicating that tuberculin testing was unnecessary to determine whether or not neonates had developed immunity to the BCG vaccine [49].

Malik et al. conducted a case-control study in Santiago, Chile, to determine if BCG immunization effectively protected young individuals against pulmonary TB. There were a total of 188 healthy controls and 68 young people who had just received a diagnosis of pulmonary TB in the trial. Results showed that vaccination effectiveness was 10%, with 13.2% of cases and 12.2% of controls not receiving the shot. The proportion of patients and controls with one, two, or three BCG scars was likewise not significantly different. According to the research, protective immunity caused by BCG vaccination might be nullified by inherited or acquired predisposing variables. Research on the effectiveness of the BCG vaccine in preventing pulmonary TB was inconclusive [50].

According to research by Bannister et al., 33 responses were observed among 71,341 Brazilian kids, with one adverse reaction every 2854 immunizations. Second-dose responses were more prevalent than first-dose reactions, and the majority of reactions were local cutaneous lesions. The research found that second-dose adverse events are very uncommon and do not impact the revaccination policy [21].

Malik et al. conducted a research in Japan that looked at the cost-benefit analysis of a BCG revaccination program implemented in 1996 for kids. The purpose of the research was to calculate the program's benefit-cost ratio and to estimate the quantity and expense of vaccines required to prevent TB cases. A total of 1.35 million elementary school children in the first grade and 1.51 million junior high school students in the first grade participated in the research. Findings indicated that over a decade, the program would save 296 TB cases, with an associated cost of $108,378 per case avoided. A pulmonary TB treatment for a 10-year-old kid would cost around $11,576 (a fraction of the cost of prevention), and the benefit-cost ratio would be 0.13 [50].

## Conclusions

The BCG vaccine is the only approved vaccine for preventing TB. Nevertheless, it does neither prevent primary infection nor the reactivation of latent pulmonary infection, which is the principal channel via which bacilli are transmitted. The level of effectiveness, on the other hand, differs depending on the setting and the age group being considered.
